# Metagenomic Insight into the Associated Microbiome in Plasmodia of Myxomycetes

**DOI:** 10.3390/microorganisms12122540

**Published:** 2024-12-10

**Authors:** Xueyan Peng, Shu Li, Wenjun Dou, Mingxin Li, Andrey A. Gontcharov, Zhanwu Peng, Bao Qi, Qi Wang, Yu Li

**Affiliations:** 1 Engineering Research Center of Chinese Ministry of Education for Edible and Medicinal Fungi, Jilin Agricultural University, Changchun 130118, China; pxy_1996@163.com (X.P.); dwj1575466@outlook.com (W.D.); li_xiaoming2022@outlook.com (M.L.); qibao3712@163.com (B.Q.); fungi966@126.com (Y.L.); 2 Hefei Mycological Valley Innovation Institute, Hefei 231131, China; ls_0830@163.com; 3 Federal Scientific Center of the East Asia Terrestrial Biodiversity, Far Eastern Branch of the Russian Academy of Sciences, 690022 Vladivostok, Russia; gontcharov@biosoil.ru; 4 Information Center, Jilin Agricultural University, Changchun 130118, China

**Keywords:** slime mold, metagenome, coexistence, core microbiome, functional redundancy

## Abstract

During the trophic period of myxomycetes, the plasmodia of myxomycetes can perform crawling feeding and phagocytosis of bacteria, fungi, and organic matter. Culture-based studies have suggested that plasmodia are associated with one or several species of bacteria; however, by amplicon sequencing, it was shown that up to 31–52 bacteria species could be detected in one myxomycete, suggesting that the bacterial diversity associated with myxomycetes was likely to be underestimated. To fill this gap and characterize myxomycetes’ microbiota and functional traits, the diversity and functional characteristics of microbiota associated with the plasmodia of six myxomycetes species were investigated by metagenomic sequencing. The results indicate that the plasmodia harbored diverse microbial communities, including eukaryotes, viruses, archaea, and the dominant bacteria. The associated microbiomes represented more than 22.27% of the plasmodia genome, suggesting that these microbes may not merely be parasitic or present as food but rather may play functional roles within the plasmodium. The six myxomycetes contained similar bacteria, but the bacteria community compositions in each myxomycete were species-specific. Functional analysis revealed a highly conserved microbial functional profile across the six plasmodia, suggesting they may serve a specific function for the myxomycetes. While the host-specific selection may shape the microbial community compositions within plasmodia, functional redundancy ensures functional stability across different myxomycetes.

## 1. Introduction

Myxomycetes are a group of amoeboid eukaryotes with complex life cycles [1], which not only have fruiting bodies that reproduce by spores, but some species form plasmodia during their trophic phase [2]. Plasmodia are motile, ranging in size from a few micrometers to over a meter [3], and contain several to thousands of synchronously dividing nuclei [4]. Plasmodia can feed on bacteria, fungi, algae, and other organic matter [5,6]. Myxomycetes are widely distributed in terrestrial ecosystems [7], and are the most species-rich taxon known in the Amoebozoa (Lado, C. (2005–2024)) [8].

Myxomycetes are a key branch for understanding the evolutionary history of eukaryotes [1,9,10,11]. Protists are the key components of food webs [12,13,14], and they interact closely and functionally with a wide range of prokaryotes [15]. Considering the ability of microorganisms to extend the evolutionary potential of their hosts [16], the diversity of eukaryotes-associated microorganisms and their functional characterization seems to be a fundamental issue. However, this issue has not been sufficiently studied [3,17]. Notably, it has been more limitedly investigated in myxomycetes [15]. According to previous studies, bacteria are an important food source for myxomycetes [5,18], and this association between myxomycetes and bacteria is not only present in the trophic phase of myxomycetes but also in the fruiting body phase [19,20,21].

According to the culture-based method, one to several bacteria were associated with one myxomycete [22]; however, by amplicon sequencing, it was shown that up to 31–52 bacteria species could be detected in one myxomycete [21,23], suggesting that the bacterial diversity associated with myxomycetes were likely to be underestimated. To fill this gap and characterize myxomycetes’ microbiota and functional traits, we performed metagenomic sequencing on the plasmodia of six myxomycetes species (*Didymium squamulosum* (Alb. and Schwein.) Fr., *D. nigripes* (Link) Fr., *Fuligo gyrosa* (Rostaf.) E. Jahn, *Badhamia melanospora* Speg., *Arcyria cinerea* (Bull.) Pers., and *Macbrideola scintillans* H.C. Gilbert.). These myxomycetes belong to several genera [24], and their trophic phases include phaneroplasmodium and aphanoplasmodium [4], which are easier to collect. We hope our data will provide new insights into the diversity and function of microbiota associated with myxomycetes.

## 2. Materials and Methods

### 2.1. Laboratory Culture of Plasmodia

Fruiting bodies of *D. squamulosum*, *D. nigripes*, *P. gyrosa*, *B. melanospora*, *A. cinerea*, and *M. scintillans* were collected in the forest at four sites (Supplementary Table S1). Morphological characteristics, including hypothallus, peridium, spore mass, lime, capillitium, columella, and stalk, were observed under a light microscope (LM, Zeiss Axio Scope A1, Oberkochen, Germany) [1]. The peridium of fruiting bodies was surface-sterilized with 75% ethanol [23]. After washing residual ethanol with sterile water, a single fruiting body was placed in a 2 mL centrifuge tube with sterile water (to ensure successful germination, ten fruiting bodies of each myxomycetes species were individually placed in centrifuge tubes for spore germination). The fruiting bodies were then crushed with sterile tweezers to release spores, preparing a spore suspension. The suspension was incubated in the dark at 23 °C. The myxamoeba were released from the germinated spores, then the spore suspension drops were placed on an oat agar medium and incubated at 23 °C in the dark [25]. The young plasmodia formed on the medium were transferred to fresh sterile oat agar medium. Upon maturation of the older plasmodia, a single, most vigorous plasmodium was selected for expansion and regular subculturing onto a fresh medium. For three months, subculturing was carried out every five days, alternating between oat and water agar mediums. The plasmodial culture design is shown in Supplementary Figure S1a.

### 2.2. DNA Extraction and Metagenomic Sequencing

Three biological replicates were included for each sample, with each biological replicate from six plasmodia cultured under parallel conditions. Plasmodia were collected into 2 mL sterile centrifuge tubes, immediately frozen in liquid nitrogen, and stored at −80 °C. Total genomic DNA was extracted from the plasmodia using the TiaNgen Plant Genomic DNA Kit (TIANGEN BIOTECH (BEIJING) Co., Ltd., Beijing, China) according to the manufacturer’s protocols. DNA purity was examined by electrophoresis on 1% agarose gel. For metagenomic library preparation, a paired-end library was constructed using Covaris M220 (Gene Company Limited, Shanghai, China) to obtain fragments with an average size of approximately 350 bp. The paired-end library was prepared using the NEXTFLEX Rapid DNA-Seq Kit (Bio Scientific, Phoenix, AZ, USA). Paired-end sequencing was performed on an Illumina Novaseq X Plus platform (Illumina, Inc., San Diego, CA, USA) at Majorbio—Pharm Technology Co., Ltd. (Shanghai, China). The experimental design is shown in Supplementary Figure S1b.

### 2.3. Quality Control and Genome Assembly

The data were analyzed on the free online Majorbio Cloud Platform. Fastp (https://github.com/OpenGene/fastp, Version 0.20.0, accessed on 3 March 2024) was used to remove adapters and low-quality reads [26]. The reads were aligned to the host genome (for *D. squamulosum*, only the sequences of *D. squamulosum* itself were removed; for other Myxomycetes, the sequences of both *D. squamulosum* and *Physarum polycephalum* Schwein. were removed as host genomes) using BWA [27] (http://bio-bwa.sourceforge.net, Version 0.7.9a, accessed on 3 March 2024) to remove contaminating reads. The genome of *D. squamulosum* has not yet been published.

### 2.4. Gene Prediction, Taxonomy, and Functional Annotation

High-quality reads were assembled into longer contigs using MEGAHIT [28] (https://github.com/voutcn/megahit, Version 1.1.2, accessed on 3 March 2024). Contigs with a length of ≥300 bp were selected and then open reading frame (ORF) prediction was performed using Prodigal [29] (https://github.com/hyattpd/Prodigal, Version 2.6.3, accessed on 3 March 2024), with genes longer than 100 bp selected for downstream analysis.

The predicted gene sequences were clustered using CD-HIT [30] (http://www.bioinformatics.org/cd-hit/, Version 4.6.1, accessed on 3 March 2024) with parameters set to 90% identity and 90% coverage to construct a non-redundant gene catalog. High-quality reads from each sample were aligned to the non-redundant gene catalogs to calculate gene abundance using SOAPaligner [31] (https://anaconda.org/bioconda/soapaligner, Version 2.21, accessed on 3 March 2024) at 95% identity.

The amino acid sequences of the non-redundant gene catalog were annotated based on the NR, COG, KEGG, and Probio probiotic databases using BLASTP Version 2.3.0 as implemented in Diamond [32] (https://github.com/bbuchfink/diamond, Version 0.8.35, accessed on 3 March 2024) with an e-value cutoff of 1 × 10^−5^ to obtain annotations for taxonomy and function. The non-redundant gene catalog was also compared against the CAZy V8 using hmmscan with an e-value cutoff of 1 × 10^−5^.

### 2.5. Statistical Analysis

Statistical analyses were performed using SPSS version 22.0. The relative abundance maps of microbial communities and Venn diagrams were generated from the corresponding taxonomic data. Alpha diversity (Chao1, Shannon and Pielou_e indices) and beta diversity distance matrices were calculated using QIIME2 [33]. The principal coordinate analysis (PCoA) and the hierarchical clustering analysis (weighted Unifrac UPGMA) were based on the Bray–Curtis distance matrices and then we used R to visualize them [34]. Differences in community composition were tested using permutational multivariate analysis of variance (PERMANOVA). Figures were generated from R version 4.2.0 [35] packages (VennDiagram [36], vegan [37], phyloseq [38], ggplot2 [39]) and the free Majorbio Cloud Platform (http://www.majorbio.com). The non-parametric factorial Kruskal–Wallis test and Dunn’s multiple comparison tests within the STAMP program (http://kiwi.cs.dal.ca/Software/STAMP, accessed on 10 August 2024) were applied to compare sample data in cases of heteroscedasticity or non-normally distributed variables. Microbial taxa (at the species level) were linked to functions using genes, and the functional contributions of microbial taxa to functional pathways were analyzed using R scripts [40]. Statistical significance was set at *p* values < 0.05.

## 3. Results

### 3.1. Associated Microbiome Accounted for a Substantial Proportion of Plasmodia

**Figure 1:**
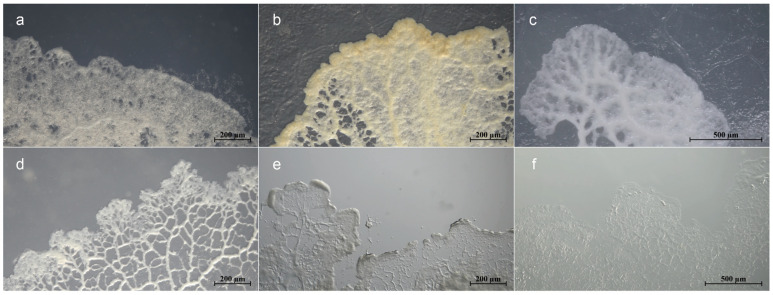
Six plasmodia of myxomycetes cultured on water agar media: phaneroplasmodium (**a**) *D. squamulosum*, (**b**) *D. nigripes*, (**c**) *F. gyrosa*, (**d**) *B. melanospora*, and aphanoplasmodium (**e**) *A. cinerea*, (**f**) *M. scintillans*.

Plasmodia-associated microbial communities of six myxomycetes (Figure 1, Table 1), were investigated by metagenomic sequencing. Following quality control on the raw reads for *D. squamulosum*, 135,298,924 high-quality clean reads were obtained (Supplementary Table S2). After removing sequences of the host genome of *D. squamulosum* [41], 27,154,536 optimized reads were retained, and the associated microbiome represented 22.27% of the holobiont (Table 1, Supplementary Table S2). As reference genome information for *D. nigripes*, *F. gyrosa*, *B. melanospora*, *A. cinerea*, and *M. scintillans* were unavailable, the genomes of *P. polycephalum* and *D. squamulosum* were used to remove sequences of the host genome. This resulted in 406,836,220 high-quality clean reads for downstream analysis (Supplementary Table S2). The proportion of associated microbiomes ranged from 46.49% to 77.40% of the holobiont in five myxomycetes’ plasmodia (Table 1).

**Figure 2:**
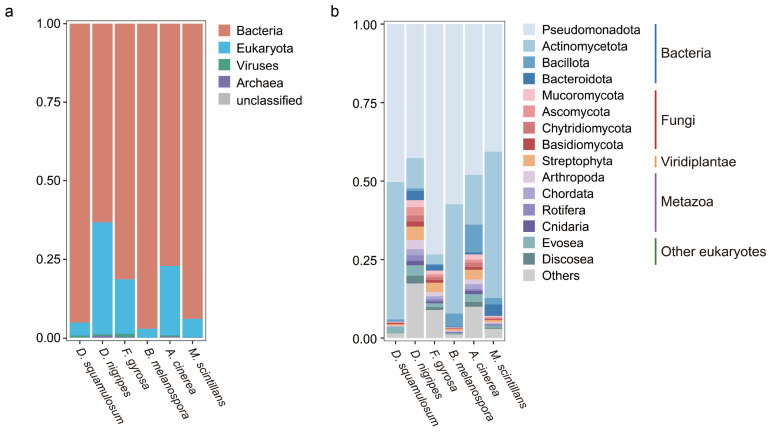
Relative abundance of microbial communities in each plasmodium. The domain level (**a**) and top 15 phylum level (**b**) are displayed at community compositions. Phylum outside the top 15 samples was assigned as “Others”.

In *D. squamulosum*, bacteria constituted the predominant group of the associated microbiome, accounting for 95.17% of the total microbiome, followed by eukaryotes, which accounted for 4.12% of the total microbial genome, of which fungi accounted for 0.81%, with the remaining 0.72% comprising viruses and archaea (Figure 2a). In the other five plasmodia, bacteria were also the most abundant group, accounting for 63.24% to 97.09% of the plasmodia-associated microbiome. Fungi, within the eukaryotic domain, represented 0.58% to 9.18% (Figure 2b). Given the overwhelming dominance of bacteria in the plasmodia, subsequent analyses focused on bacteria.

### 3.2. Bacterial Communities Associated with Plasmodia

**Figure 3:**
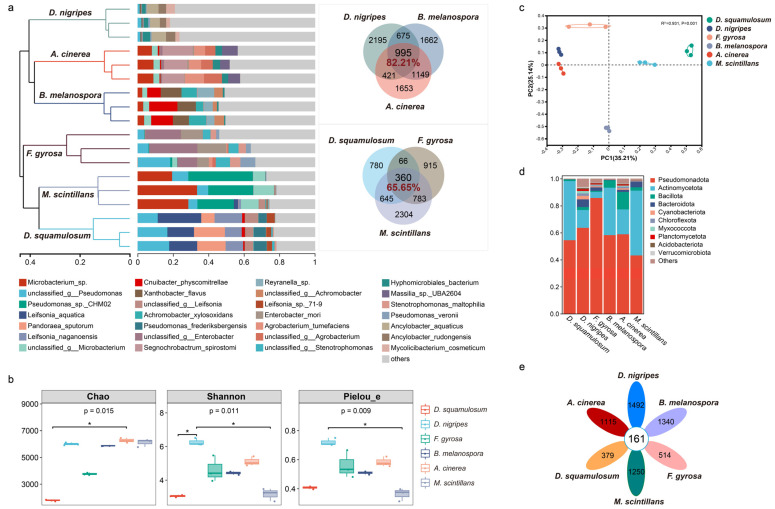
Bacterial community composition and diversity analysis of each plasmodium. (**a**) Hierarchical clustering analysis (weighted Unifrac UPGMA) and relative abundance of bacterial communities associated with each plasmodium and Venn diagrams show the number and abundance of shared and unique bacteria in each plasmodium at the genus level. (**b**) The α diversity (Chao, Shannon index, and Pielou_e) and (**c**) PCoA analysis using the Bray–Curtis distance metric showed the plasmodia bacterial communities’ diversity. (**d**) Relative abundances of the top 10 phylum levels of bacterial communities. (**e**) Venn diagram shows the shared and unique bacteria at the species level in each plasmodia sample. The differences were considered significant when *p* values < 0.05. *: *p* < 0.05.

The bacterial community composition at the species level within different myxomycete plasmodia remains distinct and exhibits its own unique bacterial community composition under identical nutrient sources and environmental conditions (Figure 3a). Alpha diversity indices (Figure 3b), including Chao1, Shannon, and Pielou_e indices, and principal coordinate analysis (PERMANOVA, R^2^ = 0.93135) (Figure 3c) supported this observation (*p* < 0.05). However, the bacteria community composition at the phylum level was similar in six plasmodia (Figure 3d), and most bacteria belonged to two phyla.

In plasmodia of *D. squamulosum*, *Leifsonia aquatica* (*ex* Leifson 1962) Evtushenko et al. 2000, *Pseudomonas* sp., *Pandoraea sputorum* Coenye et al. 2000, and *L. naganoensis* Suzuki et al. 2000 accounted for 58.54% of all bacterial communities (Figure 3a). This pattern of dominant bacteria, where a few species of bacteria represented a fairly high abundance of the microbiome, was also observed in the plasmodia-associated bacterial communities with the other five myxomycetes species. Bacterial communities in the six myxomycetes formed two clusters. *D. nigripes* and *B. melanospora*, which belong to Physarales, and *A. cinerea*, which belong to Trichiales, were clustered together; *D. squamulosum* and *F. gyrosa* which belong to Physarales and *M. scintillans,* which belong to Stemonitidales, were clustered together. *D. squamulosum* and *D. nigripes* belong to the same family of Didymiaceae, but the bacterial communities in these two myxomycetes were clustered into two separate clusters.

Even distantly related myxomycetes (at the order level) shared core bacteria (Figure 3a, e). *D. nigripes*, *B. melanospora*, and *A. cinerea* shared 995 bacteria and accounted for 82.21% of the total bacterial community; *D. squamulosum*, *D. nigripes,* and *M. scintillans* shared 360 bacteria and accounted for 65.65% of the total bacterial community. All six species shared 161 bacterial species and accounted for 50.56% of all bacteria communities (Figure 3e), while unique bacterial species within each myxomycete ranged from 0.79–4.54% only. Despite this shared core microbiota, substantial differences were observed in the abundance of dominant bacterial communities at the species level within each myxomycete. These results showed that while the six myxomycetes harbored similar bacterial species, their species composition and relative abundance were species-specific.

### 3.3. Microbiota Phenotypes and Functional Prediction of Bacterial Communities Associated with Plasmodia

**Figure 4:**
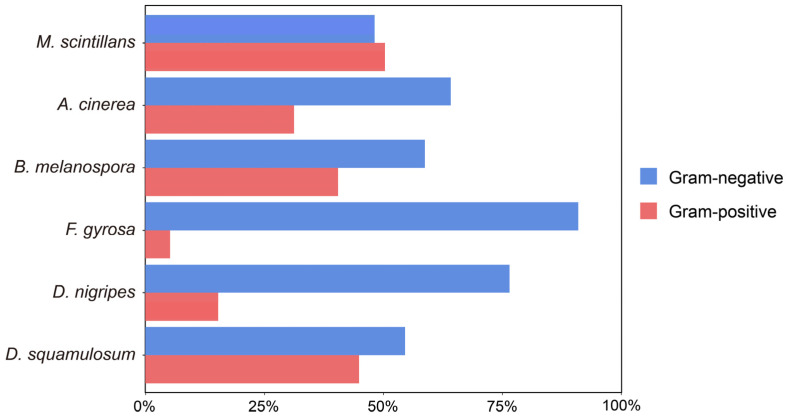
Relative abundance of Gram-positive/Gram-negative bacteria in each plasmodium.

Gram-negative bacteria were predominant in the plasmodia of all myxomycetes species. However, a considerable proportion of Gram-positive bacteria were also observed in *D. squamulosum*, *B. melanospora*, *A. cinerea*, and *M. scintillans*, which reached up to 50.50% (Figure 4).

**Figure 5:**
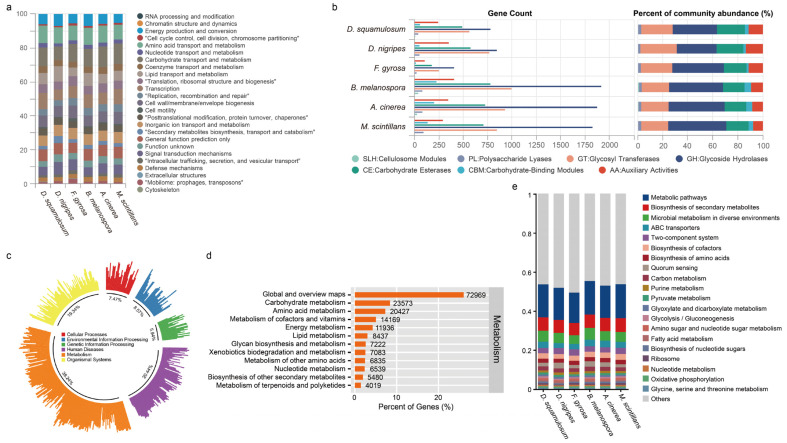
Functional analysis of plasmodia-associated bacteria. (**a**) A comparison of the top 25 COG functional categories in the six plasmodia. (**b**) Gene count and relative abundance of CAZy class categories. (**c**) Functional KEGG level 1 and (**d**) KEGG level 2 pathway descriptions, and (**e**) relative abundance of top 20 KEGG level 3 pathway categories.

The functional capabilities of the endo-plasmodial bacterial communities were elucidated using the COG, CAZy, and KEGG databases. The COG annotation revealed that bacteria in six myxomycetes exhibited various functional activities, with a predominance of proteins associated with metabolism, particularly amino acid and carbohydrate metabolism (Figure 5a). The carbohydrate-active enzymes were primarily enriched in glycoside hydrolases (GHs), glycosyltransferases (GTs), and carbohydrate esterases (CEs) at all six plasmodia of myxomycetes, as shown by gene comparisons against the CAZy database (Figure 5b). The total nonredundant protein-coding genes were primarily represented by the function “metabolism” (38.24%) (Figure 5c). The most prevalent KEGG level 2 subcategories were within the “metabolism category”, “global and overview maps”, “carbohydrate metabolism”, and “amino acid metabolism” (Figure 5d). The endo-plasmodial bacterial communities of six myxomycetes species exhibited remarkable functional capabilities similarity in all three analyses using COG, CAZy, and KEGG databases (Figure 5e, Supplementary Figures S2–S4). Correlating bacterial species with carbohydrate-hydrolyzing enzymes, protein functions, and metabolic pathways revealed that numerous bacterial members shared similar functions, albeit with varying contributions to each function.

**Figure 6:**
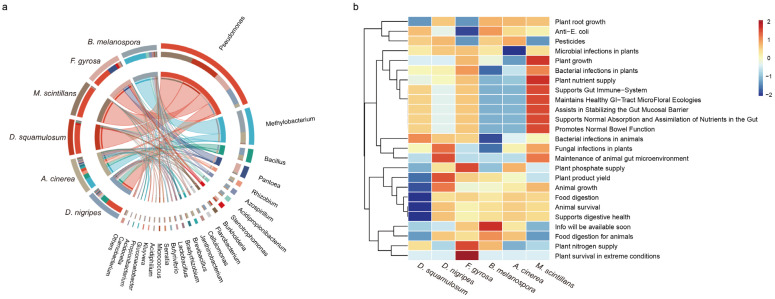
Composition and functional analysis of probiotics in six myxomycetes. Relative abundance of probiotics at genus level (**a**) and top 25 of functional composition of probiotics (**b**) was exhibited in each plasmodium.

Additionally, we evaluated the probiotic potential of plasmodia using the Probio database. Probiotic bacteria found in plasmodia, such as *Pseudomonas* spp., *Methylobacterium* spp., *Bacillus* spp., *Pantoea* spp., *Rhizobium* spp., etc. (Figure 6a), were primarily associated with plant growth, nutrient absorption of plant and animal, and promotion of the intestinal immune system (Figure 6b), which implied that those probiotic bacteria among the plasmodia could be contributors to the host’s growth and development.

## 4. Discussion

Microorganisms are ubiquitous and can be found anywhere, including inside organisms [42], forming symbiotic communities known in nearly all protists’ taxonomic groups [15]. However, there is still little information on the magnitude of abundance that symbiotic microbes account for in the host. In sponges, the harbored microbes account for up to 40% of their volume [43]; but only 0.2% and 0.09% in adult female mites and eggs [44]. To our knowledge, there are no metagenome-based reports on symbiote abundance among protists. Our results showed that microbes accounted for 22.27% of the total genome of the *D. squamulosum* plasmodia, whereas the microbes in the other five plasmodia accounted for 46.49–77.40% of the total host genome. The metagenome data of *D. squamulosum* were analyzed using its well-assembled reference genome data. In contrast, no such genome sequences were available for the other five myxomycetes species; therefore, we used the genomes of both *D. squamulosum* and *P. polycephalum* instead. Consequently, the result of 22.27% may be more accurate. Such a high abundance of microbes in myxomycetes implied that these microbes may have a functional role in the plasmodia rather than being merely parasitic or present as food.

Several studies have indicated that protists live in predation, symbiosis, or parasitism with bacteria, fungi, plants, and archaea [1,2,15,45,46]. However, the extent of these relationships remains poorly studied. Myxomycetes have been reported to establish more stable associations with bacteria [22,47], and are also found to have a far beyond predator-prey relationship with diverse microbes, including fungi [48,49,50,51] and algae [6,52,53,54]. In our study, fungi, viridiplantae, metazoa, viruses, and archaea DNA sequences were also detected in myxomycetes besides bacteria. In *D. squamulosum*, the host genome was sufficiently removed because it has a well-assembled reference genome; the presence of metazoan and viridiplantae sequences is most likely due to DNA fragment contamination. Although the oat agar medium was strictly sterilized and most DNA in the medium was disturbed, autoclaving under the standard conditions of 121 °C for 15 min did not sufficiently remove all DNA [55] and can also be detected. The presence of fungi and virus sequences can result from DNA fragment contamination, or *D. squamulosum* harbors these microbes. Some microbes might exhibit dormant states or be packaged within the protist that escaped sterilization and persisted in a quiescent state within the plasmodia, which might become active only under specific environmental conditions [56,57]; similar situations could explain their presence and low abundance in myxomycetes. Moreover, some metazoan, prokaryote (bacterial and archaeal), fungal, and unicellular algal sequences were identified as potential horizontal gene transfer (HGT) candidates within protists [58]. We do not dismiss these possibilities, and future research based on culturing and genomics should be performed to establish the existence of eukaryotic microbes in myxomycetes. As reference genome information for the other five myxomycetes was unavailable, the presence of DNA sequences from microbes other than bacteria, especially for these eukaryotic DNA sequences, is more likely due to insufficient removal of the host genome.

One common feature of the plasmodia-associated bacterial communities is that dominant bacteria exist, and each myxomycetes species harbors its specific dominant bacteria. Even distantly related myxomycetes shared core bacteria; their species composition and relative abundance are species-specific. Due to the feeding habits of plasmodia, the source of bacteria within them is likely related to their surrounding collection site and substrates, which is well-documented in animal systems [59,60,61,62]. However, hierarchical clustering analysis revealed that the endo-plasmodial bacterial community similarity is not correlated with their taxonomic similarity, geographic proximity, or similar substrate. Although the bacteria community in each myxomycete is significantly different, there are striking similarities in the functional composition of bacteria, which implies that they may serve a specific function for the myxomycetes, corroborating a previous report [23]. Functional redundancy may be a plausible explanation for different populations of dominant bacteria showing striking similarities in functional composition [63,64]. Functional redundancy is widespread in microbial systems [63,65]. Hundreds of microorganisms capable of oxidizing hydrogen coexist in groundwater [66], and many of the metabolic traits of intestinal microorganisms are functionally redundant while variable in their composition [67].

There could be another reason the core bacterial flora in plasmodia are similar, but the dominant bacteria are species-specific. The bacterial composition of different myxomycetes under the same nutrient conditions varies significantly, and the host myxomycetes itself may play a central role in the establishment of the bacterial community, which may selectively enrich or exclude specific bacteria by secreting different compounds, providing different microenvironments, and so on. Bacterial diversity in *D. squamulosum* is substantially lower than in the other studied myxomycetes; the minimal diversity in *D. squamulosum* could be attributed to sufficiently removing the host genome.

In contrast to the general notion that Gram-negative bacteria are the overwhelming majority of the myxomycetes-associated bacteria [5,22,23]. We revealed that Gram-positive bacteria account for a significant proportion of bacterial communities in *D. squamulosum*, *B. melanospora*, *A. cinerea*, and *M. scintillans*. Differences in species and individuals may be one of the reasons for the discrepancies [68], and in addition, fluctuations in function-centered taxa over time and changes in the surrounding environment may also explain the high proportion of Gram-positive bacteria in some species [65,69,70,71].

## 5. Conclusions

The plasmodia harbor diverse microbial communities, including eukaryotes, viruses, archaea, and the dominant bacteria. The associated microbiomes represented more than 22.27% of the plasmodia genome, suggesting that these microbes may not merely be parasitic or present as food but rather may play functional roles within the plasmodium. The six myxomycetes contained similar bacteria, but the bacteria community compositions in each myxomycete were species-specific. Functional analysis revealed a highly conserved microbial functional profile across the six plasmodia, suggesting they may serve a specific function for the myxomycetes. While the host-specific selection may shape the microbial community compositions within plasmodia, functional redundancy ensures functional stability across different myxomycetes.

## Figures and Tables

**Table 1:** The percentage of the associated microbiome in total reads of each sample.

Order	Family	Genus	Species	Percent of Associated Microbiome in Total Reads (%)
Physarales	Didymiaceae	*Didymium*	*D. squamulosum*	22.27
			*D. nigripes*	46.49
	Physaraceae	*Fuligo*	*F. gyrosa*	48.7
		*Badhamia*	*B. melanospora*	48.17
Trichiales	Arcyriaceae	*Arcyria*	*A. cinerea*	54.30
Stemonitidales	Stemonitidaceae	*Macbrideola*	*M. scintillans*	77.40

## Data Availability

The raw dataset of metagenomic generated for this study is available in the NCBI Sequence Read Archive under accession number PRJNA1138926. The genomic datasets presented of *Didymium squamulosum* are not readily available because the data are part of an ongoing study.

## Supplementary Materials

The following supporting information can be downloaded at: https://www.mdpi.com/article/10.3390/microorganisms12122540/s1, Table S1: Information for each myxomycetes species. Table S2: Raw data and alpha diversity indexes in each sample. Figure S1: Schematic diagram of experimental design. (a) Laboratory culture of plasmodia. (b) Sample protocols and metagenomic sequencing of plasmodia. Figure S2: Relationship of top 10 COG function categories and top 10 bacteria in each plasmodium. Figure S3: Relationship of top 10 CAZy family categories and top 10 bacteria in each plasmodium. Figure S4: Relationship of top 10 KEGG pathway categories and top 10 bacteria in each plasmodium.
