# Supplementary material for: Metagenomic Insight into the Associated Microbiome in Plasmodia of Myxomycetes
Source: Microorganisms. 2024 Dec 10;12(12):2540. doi: 10.3390/microorganisms12122540 (PMC11677963; doi:10.3390/microorganisms12122540)
Supplement: Supplementary file 1 [file microorganisms-12-02540-s001.zip › Supplementary Files/Supplementary Figure 2.pdf]

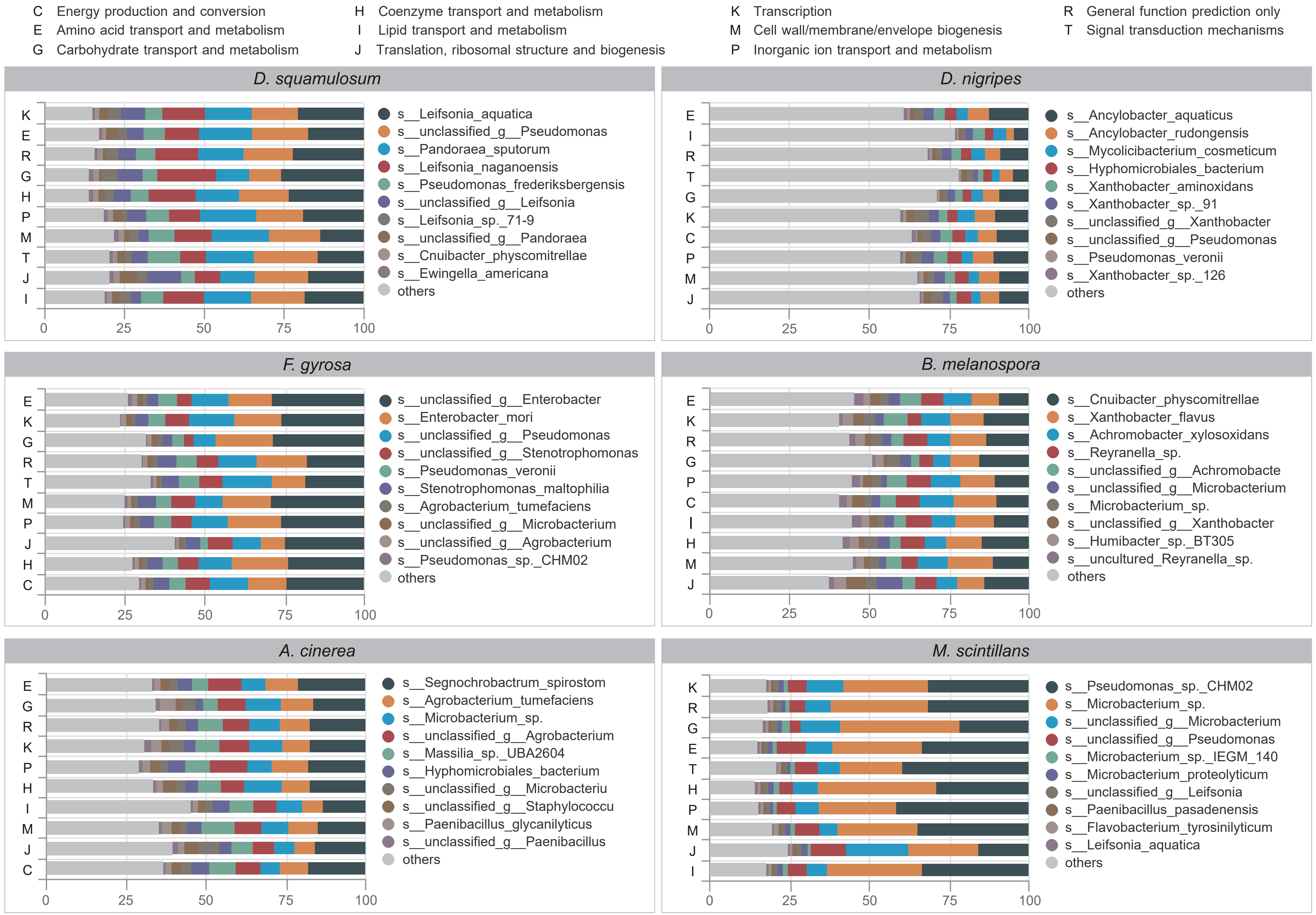

C Energy production and conversion
E Amino acid transport and metabolism
G Carbohydrate transport and metabolism
H Coenzyme transport and metabolism
I Lipid transport and metabolism
J Translation, ribosomal structure and biogenesis
K Transcription
M Cell wall/membrane/envelope biogenesis
P Inorganic ion transport and metabolism
R General function prediction only
T Signal transduction mechanisms
D. squamulosum
K
E
R
G
H
P
M
T
J
I
0 25 50 75 100
s__Leifsonia_aquatica
s__unclassified_g__Pseudomonas
s__Pandoraea_sputorum
s__Leifsonia_naganoensis
s__Pseudomonas_frederiksbergensis
s__unclassified_g__Leifsonia
s__Leifsonia_sp._71-9
s__unclassified_g__Pandoraea
s__Cnuibacter_physcomitrellae
s__Ewingella_americana
others
D. nigripes
E
I
R
T
G
K
C
P
M
J
0 25 50 75 100
s__Ancylobacter_aquaticus
s__Ancylobacter_rudongensis
s__Mycolicibacterium_cosmeticum
s__Hyphomicrobiales_bacterium
s__Xanthobacter_aminoxidans
s__Xanthobacter_sp._91
s__unclassified_g__Xanthobacter
s__unclassified_g__Pseudomonas
s__Pseudomonas_veronii
s__Xanthobacter_sp._126
others
F. gyrosa
E
K
G
R
T
M
P
J
H
C
0 25 50 75 100
s__unclassified_g__Enterobacter
s__Enterobacter_mori
s__unclassified_g__Pseudomonas
s__unclassified_g__Stenotrophomonas
s__Pseudomonas_veronii
s__Stenotrophomonas_maltophilia
s__Agrobacterium_tumefaciens
s__unclassified_g__Microbacterium
s__unclassified_g__Agrobacterium
s__Pseudomonas_sp._CHM02
others
B. melanospora
E
K
R
G
P
C
I
H
M
J
0 25 50 75 100
s__Cnuibacter_physcomitrellae
s__Xanthobacter_flavus
s__Achromobacter_xylosoxidans
s__Reyranella_sp.
s__unclassified_g__Achromobacte
s__unclassified_g__Microbacterium
s__Microbacterium_sp.
s__unclassified_g__Xanthobacter
s__Humibacter_sp._BT305
s__uncultured_Reyranella_sp.
others
A. cinerea
E
G
R
K
P
H
I
M
J
C
0 25 50 75 100
s__Segnochrobactrum_spirostom
s__Agrobacterium_tumefaciens
s__Microbacterium_sp.
s__unclassified_g__Agrobacterium
s__Massilia_sp._UBA2604
s__Hyphomicrobiales_bacterium
s__unclassified_g__Microbacteriu
s__unclassified_g__Staphylococcu
s__Paenibacillus_glycanilyticus
s__unclassified_g__Paenibacillus
others
M. scintillans
K
R
G
E
T
H
P
M
J
I
0 25 50 75 100
s__Pseudomonas_sp._CHM02
s__Microbacterium_sp.
s__unclassified_g__Microbacterium
s__unclassified_g__Pseudomonas
s__Microbacterium_sp._IEGM_140
s__Microbacterium_proteolyticum
s__unclassified_g__Leifsonia
s__Paenibacillus_pasadenensis
s__Flavobacterium_tyrosinilyticum
s__Leifsonia_aquatica
others
