# Supplementary figures and images for: Metagenomic Insight into the Associated Microbiome in Plasmodia of Myxomycetes
Source: Microorganisms. 2024 Dec 10;12(12):2540. doi: 10.3390/microorganisms12122540 (PMC11677963; doi:10.3390/microorganisms12122540)

a

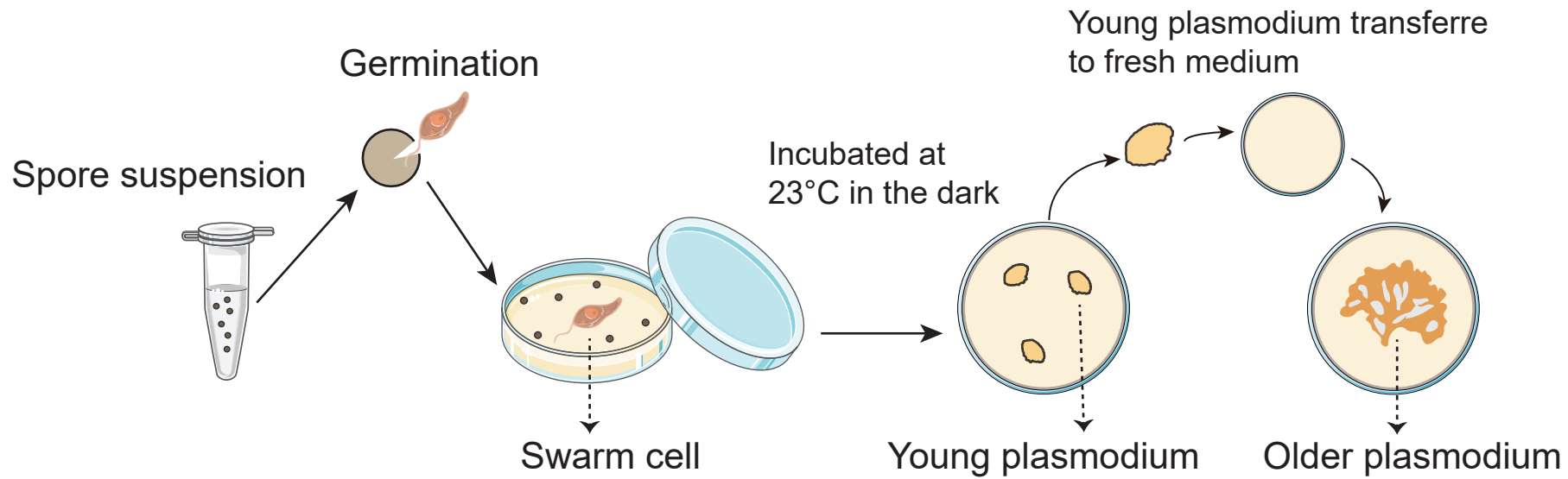

b

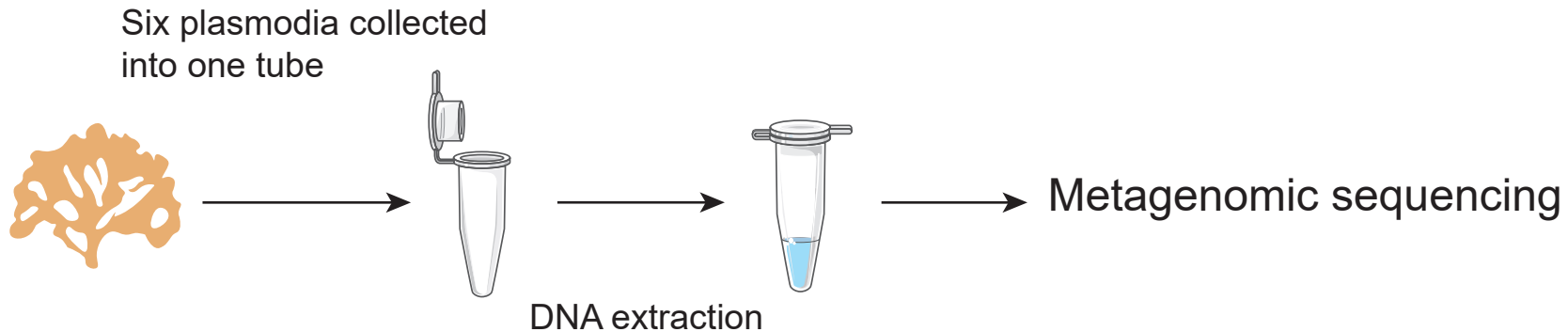

Supplement: Supplementary file 1 [file microorganisms-12-02540-s001.zip › Supplementary Files/Supplementary Figure 1.pdf]

*D. squamulosum*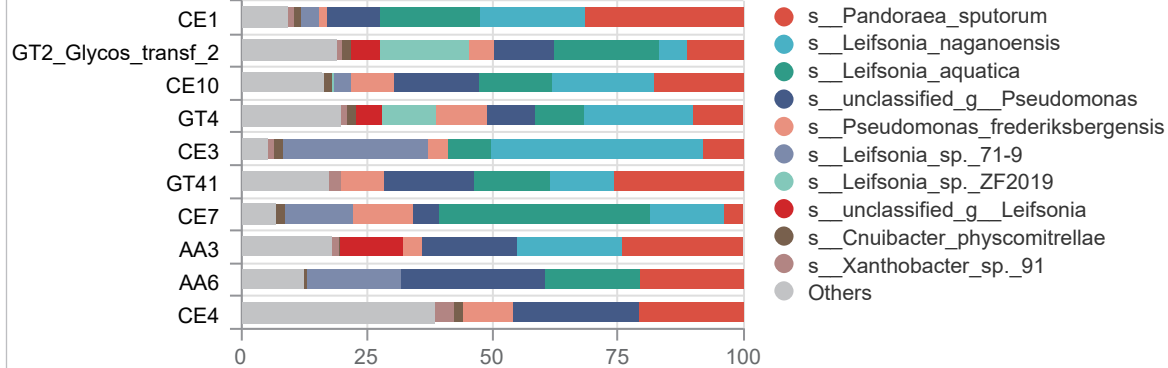*D. nigripes*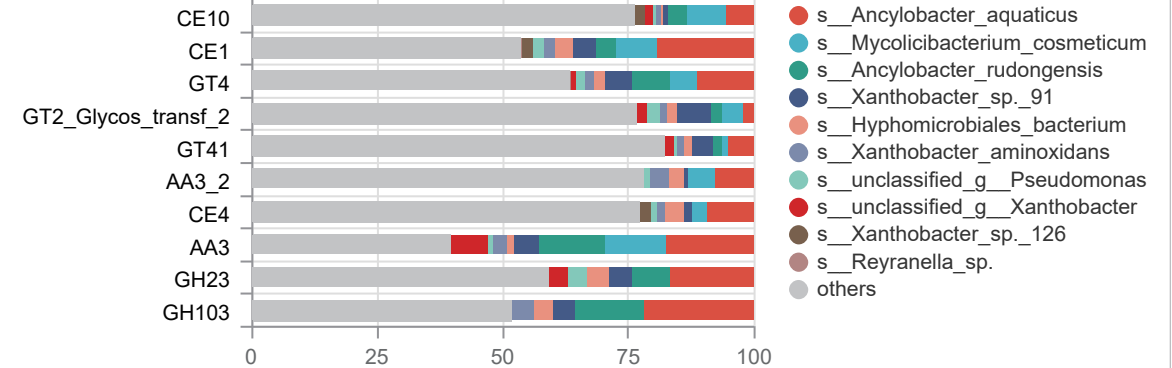*F. gyrosa*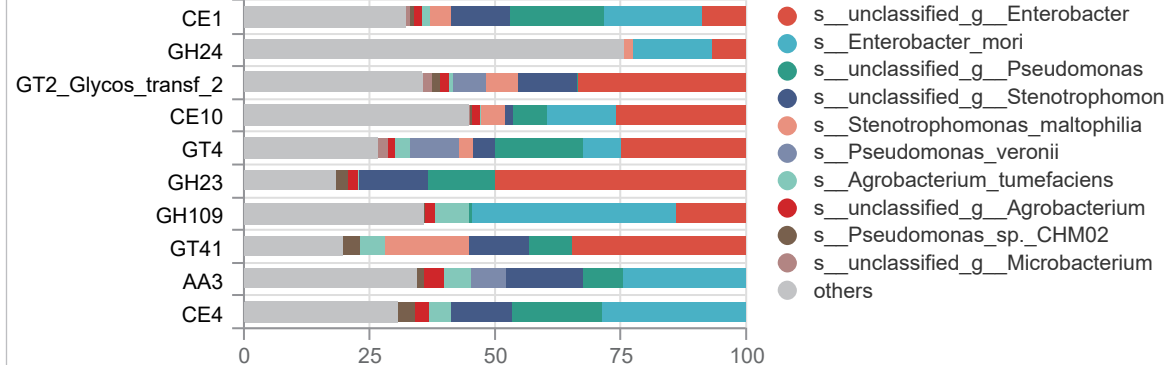*B. melanospora*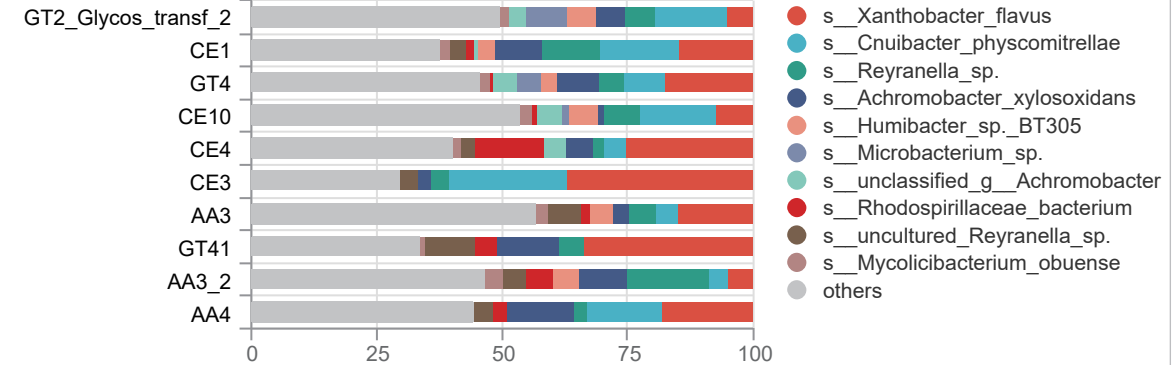*A. cinerea*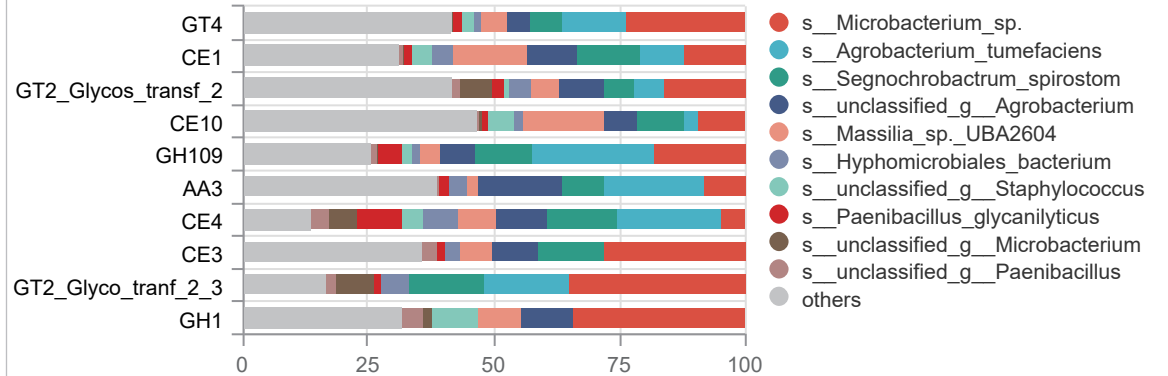*M. scintillans*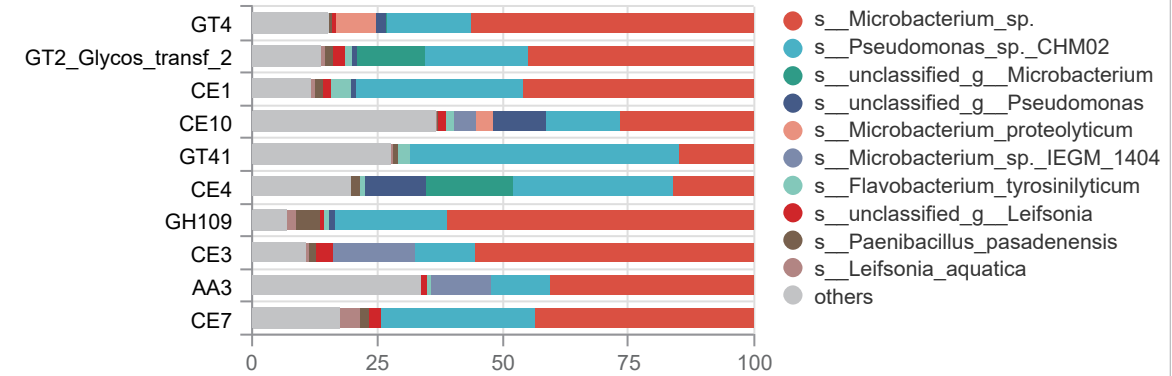

Supplement: Supplementary file 1 [file microorganisms-12-02540-s001.zip › Supplementary Files/Supplementary Figure 3.pdf]

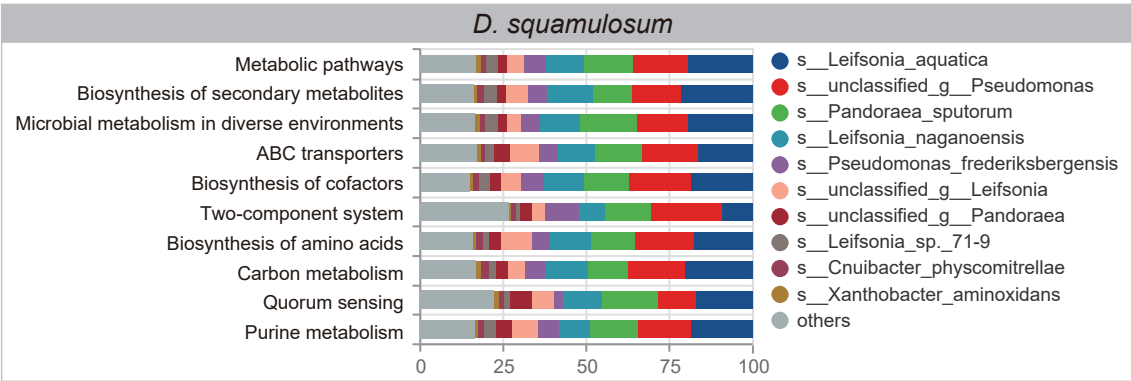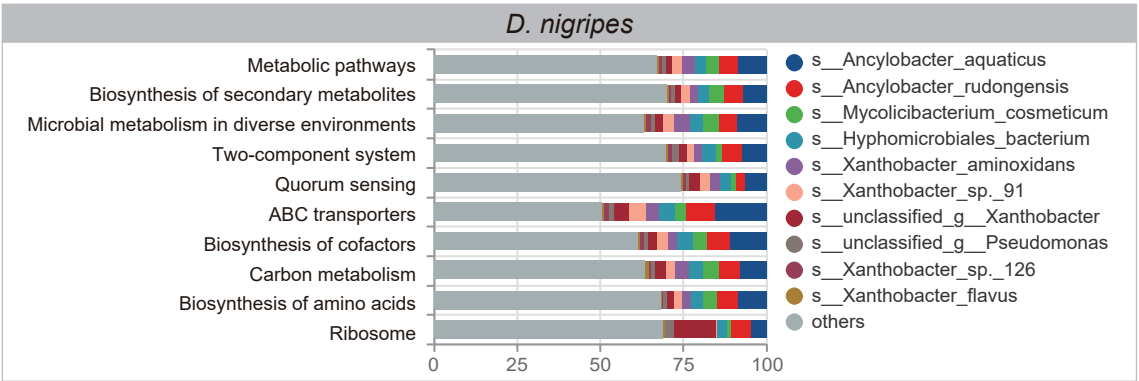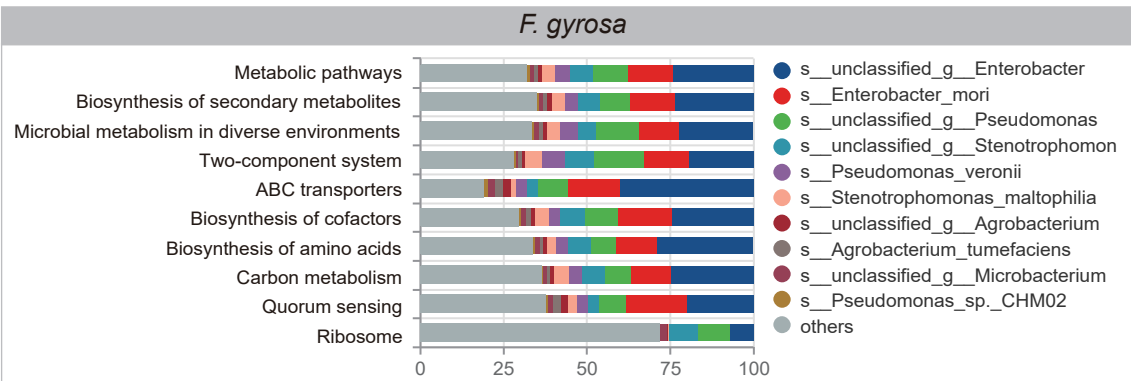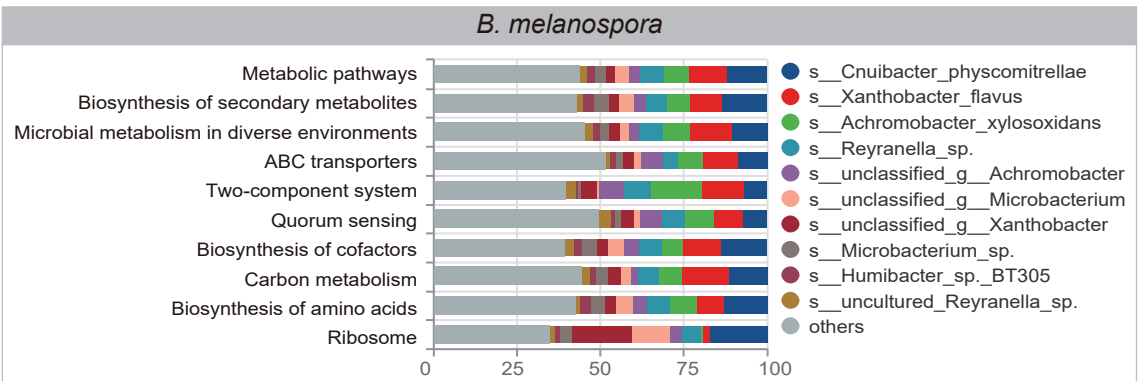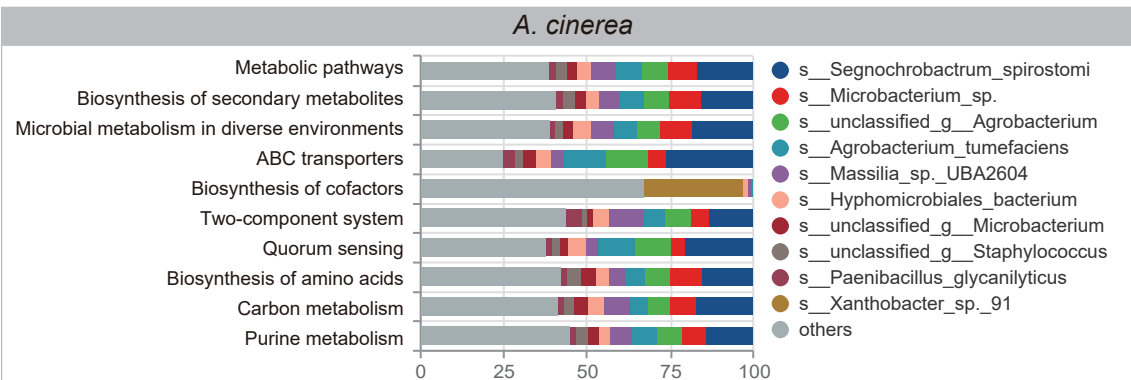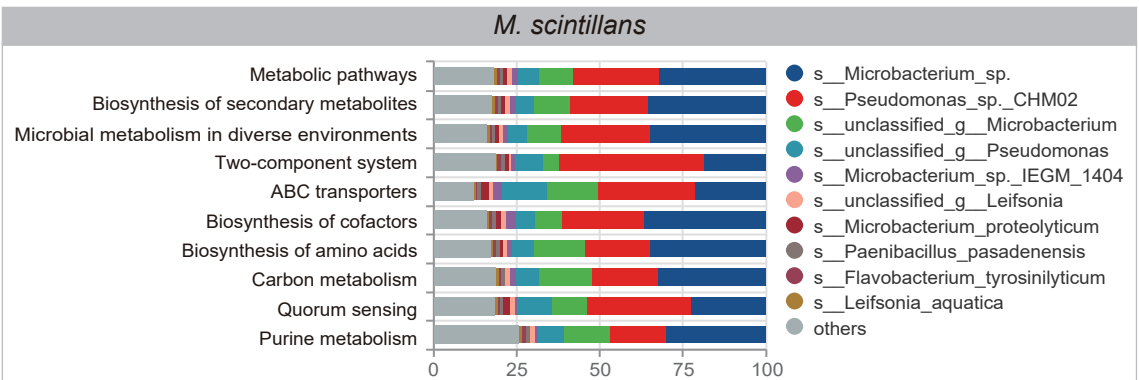

Supplement: Supplementary file 1 [file microorganisms-12-02540-s001.zip › Supplementary Files/Supplementary Figure 4.pdf]
